# German Cancer Consortium (DKTK) – A national consortium for translational cancer research

**DOI:** 10.1002/1878-0261.12430

**Published:** 2019-01-09

**Authors:** Stefan Joos, Dirk M. Nettelbeck, Anette Reil‐Held, Katja Engelmann, Alexandra Moosmann, Angelika Eggert, Wolfgang Hiddemann, Mechthild Krause, Christoph Peters, Martin Schuler, Klaus Schulze‐Osthoff, Hubert Serve, Wolfgang Wick, Josef Puchta, Michael Baumann

**Affiliations:** ^1^ German Cancer Consortium (DKTK) German Cancer Research Center (DKFZ) Heidelberg Germany; ^2^ German Cancer Research Center (DKFZ) Heidelberg Germany; ^3^ Charité Berlin Germany; ^4^ Ludwig‐Maximilians‐Universität München München Germany; ^5^ University Cancer Center Dresden Germany; ^6^ Comprehensive Cancer Center Freiburg (CCCF) Germany; ^7^ West German Cancer Center University Hospital Essen Germany; ^8^ Medical Faculty Comprehensive Cancer Center Tübingen Germany; ^9^ University Cancer Center University Hospital Frankfurt (UCT) Germany; ^10^ National Center of Tumor Diseases (NCT) Heidelberg Germany; ^11^ Heidelberg University Hospital Heidelberg Germany

**Keywords:** Germany, multisite cooperation, personalized oncology, research consortium, translational cancer research

## Abstract

The German Cancer Consortium (‘Deutsches Konsortium für Translationale Krebsforschung’, DKTK) is a long‐term cancer consortium, bringing together the German Cancer Research Center (DKFZ), Germany's largest life science research center, and the leading University Medical Center‐based Comprehensive Cancer Centers (CCCs) at seven sites across Germany. DKTK was founded in 2012 following international peer review and has positioned itself since then as the leading network for translational cancer research in Germany. DKTK is long term funded by the German Ministry of Research and Education and the federal states of each DKTK partner site. DKTK acts at the interface between basic and clinical cancer research, one major focus being to generate suitable multisite cooperation structures and provide the basis for including higher numbers of patients and facilitate effective collaborative forward and reverse translational cancer research. The consortium addresses areas of high scientific and medical relevance and develops critical infrastructures, for example, for omics technologies, clinical and research big data exchange and analysis, imaging, and clinical grade drug manufacturing. Moreover, DKTK provides a very attractive environment for interdisciplinary and interinstitutional training and career development for clinician and medical scientists.

**Abbreviations**
AIO: Arbeitsgemeinschaft Internistische Onkologie (Working Group Internistic Oncology)
CCC: Comprehensive Cancer Center
CCP: Clinical Communication Platform
CT: computed tomography
DKFZ: Deutsches Krebsforschungszentrum (German Cancer Research Center)
DKTK: Deutsches Konsortium für Translationale Krebsforschung (German Cancer Consortium)
DZD: Deutsches Zentrum für Diabetesforschung (German Center for Diabetes Research)
DZG: Deutsche Zentren der Gesundheitsforschung (German Centers for Health Research)
DZHK: Deutsches Zentrum für Herz‐Kreislauf‐Forschung (German Center for Cardiovascular Research
DZIF: Deutsches Zentrum für Infektionsforschung (German Center for Infection Research)
DZL: Deutsches Zentrum für Lungenforschung (German Center for Lung Research)
DZNE: Deutsches Zentrum für Neurodegenerative Erkrankungen (German Center for Neurodegenerative Diseases)
EANO: European association of neuro‐oncology
EGFR: epidermal growth factor receptor
FISH: fluorescent in situ hybridization
GMP: Good Manufacturing Practice
HNSCC: Head and Neck Squamous‐Cell Carcinoma
IIT: investigator‐initiated Trial
INFORM: INdividualized Therapy FOr Relapsed Malignancies in Childhood
IT: information technology
MASTER: Molecularly Aided Stratification for Tumor Eradication
MRI: magnetic resonance imaging
N2M2: NCT Neuro Master Match, a clinical (umbrella) study
NCT: Nationale Centrum für Tumorerkrankungen (National Center for Tumor Diseases)
NGS: Next‐generation Sequencing
PET: positron emission tomography
PSMA: prostate‐specific membrane antigen
RadPlanBio: RadiationDosePlan‐Image/Biomarker‐Outcome‐platform
SAB: Scientific Advisory Board
SoO: DKTK School of Oncology
WHO: World Health Organization

## 1. Background

Strategies to improve the impact of cancer research for patients have been widely discussed in Germany and Europe over the past two decades. One factor contributing to limited progress is the complexity of the disease cancer, with enormous biological heterogeneities between different tumor classes, tumors within each class in different patients, in each individual tumor (or its metastases) and during time of tumor progression and therapy. Another reason is that translating findings from basic research into the clinic is limited by structural deficits in facilitating interactions between basic and clinical research. Combatting these problems requires a critical mass of patients, resources, and infrastructures that can only be achieved through large‐scale cooperation which overcome the problem of fragmentation of cancer care and cancer research.

### 1.1. The German Centers for Health Research

In order to improve innovative translational research for the most widespread diseases and to bundle nationwide research activities in the federal system of Germany, six German Centers for Health Research (DZGs) were founded between 2009 and 2012. These are the German Centers for Neurodegenerative Disease (DZNE, 2009), Diabetes Research (DZD, 2009), Infection Research (DZIF, 2012), Lung Research (DZL, 2011), Heart and Vascular Research (DZHK, 2011), and Translational Cancer Research (DKTK, 2012). Each DZG constitutes a network of one of the National Health Research Centers organized within the Helmholtz Association, large university and nonuniversity biomedical research centers and leading university medical centers. Together, the DZGs form an integrated interdisciplinary and interinstitutional network in Germany, involving two thirds of the German university medical centers and all national as well as some other academic research institutions. Strong cooperation and state‐of‐the‐art infrastructures provide an excellent environment for translational and clinical research. The successful development of the DZGs was underscored by recent international reviews and the evaluation by the German Science Council, an advisory body to the government, concluding that the DZG network *created the prerequisites for an improved and accelerated translational research of highly relevant disease entities in Germany*.

### 1.2. DKTK: Structure and governance

**Figure 1:**
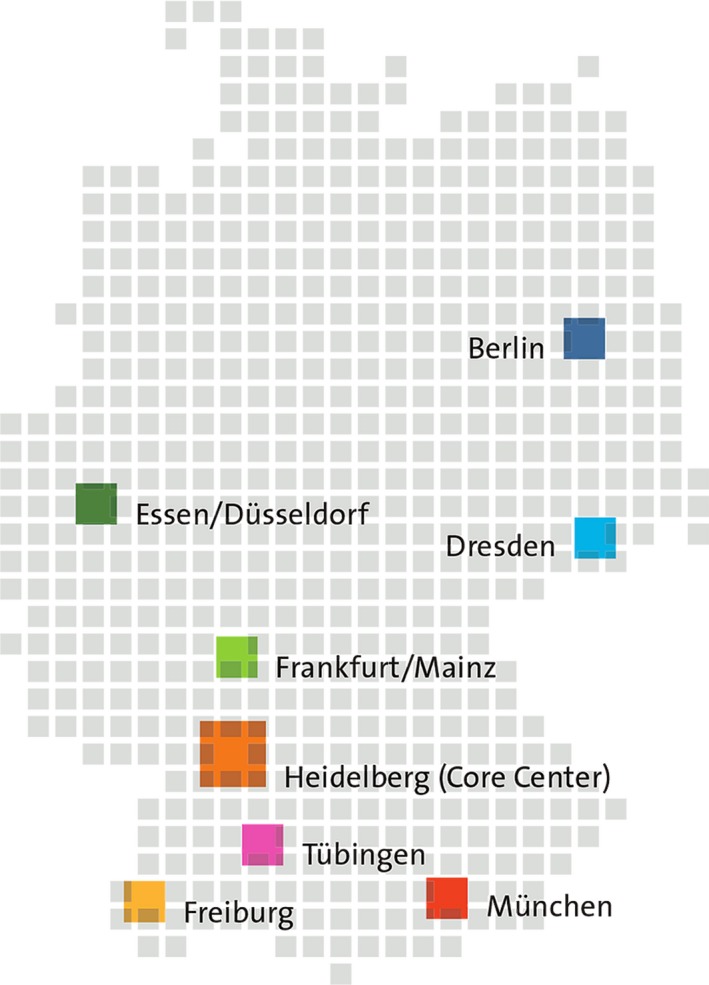
The DKTK core center and partner sites.

The members of the DKTK consortium were selected in a multistep procedure by an international expert panel following a competitive call by the German Federal Ministry of Education and Research. In addition to the DKFZ as DKTK's core center and the National Center for Tumor Diseases (NCT) in Heidelberg, the Comprehensive Cancer Centers (CCCs) at the University Medical Centers in Berlin, Dresden, Essen (together with partners from Düsseldorf), Frankfurt (together with partners from Mainz), Freiburg, Tübingen, and Munich with its two university medical centers were selected (Fig. 1). All partner sites are engaged in broad portfolios of translational and clinical cancer research, with partner site‐specific research and clinical profiles providing a solid basis for complementarity.

The DKTK's annual budget amounts to 28 Mio. Euro, 90% of which is financed by the German Federal Government through the Ministry of Education and Research, and 10% by the federal states of the participating DKTK partner sites. To guarantee its legal and financial structure as well as its long‐term sustainability, DKTK has been constituted as a foundation under public law represented by DKFZ. An important consequence of this structure is that DKTK is funded on a long‐term, institutional basis. The governance structure includes a Steering Committee that represents all member sites by their respective spokesperson, an international Scientific Advisory Board (SAB), and the Foundation Council, comprised of a representative of the German Federal Ministry of Education and Research and a representative of each of the seven German states. A DKTK coordinating bureau, which was established at the core center DKFZ in Heidelberg, supports and manages all scientific, administrative, and financial activities and processes, cooperating closely with the DKFZ administration department and local DKTK coordinators at all partner sites.

## 2. DKTK: an integrated network for multicenter translational research

The aim of DKTK is to provide an internationally recognized multicenter framework for innovative and highly competitive research in translational oncology. Special emphasis is placed on exploiting synergies by applying intelligent networking, bundling interdisciplinary expertise and critical mass from all partner sites, and strengthening the research profiles at the individual University Medical Centers. The establishment of new professorships, young investigator groups, and cutting‐edge infrastructure strongly support this endeavor.

### 2.1. Site‐overarching research programs

The DKTK acts at the interface of basic and clinical cancer research. Specific themes are addressed within currently five research programs, overall covering a number of innovative discovery research themes, forward and reverse preclinical programs, and early clinical studies (Table 1). DKTK thus contributes to several highly relevant areas of modern cancer research, including identification of molecular biomarkers for tumor classification, risk‐adapted stratification and new treatment paradigms, elucidation of individualized combination therapies and novel diagnostic and therapeutic approaches, such as immunotherapy strategies beyond checkpoint inhibition, and the development of personalized stratification strategies in radiation oncology and imaging.

**Table 1:** The DKTK's translational research programs

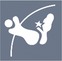	Exploitation of Oncogenic Mechanisms	Unraveling of mechanisms of tumor resistance and target identification for triggering and feeding of innovative translational pipelines
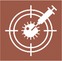	Molecularly Targeted Therapy	Preclinical validation of targets and combination therapies toward the initiation of clinical proof‐of‐concept studies as well as reverse translation
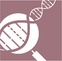	Molecular Diagnostics, Early Detection, and Biomarker Development	Development of new techniques and definition of standards for tumor diagnostics and patient treatment stratification as well as robust biomarkers relevant for early detection and the prediction of treatment response
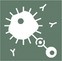	Cancer Immunotherapy	Development and exploration of personalized tumor vaccination, new therapeutic antibodies, and advanced cellular therapeutics. Combination therapies and immunomonitoring strategies are explored together with the other programs
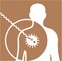	Radiation Oncology and Imaging	Preclinical and clinical studies of particle therapy and biological individualization of radiotherapy based on innovative biomarkers. Development of novel imaging biomarkers for PET, MRI, and CT and on pharmaceutical‐based theranostic approaches

### 2.2. Competitive joint funding program

The competitive Joint Funding Program with annual calls represents a key element of DKTK to trigger and expand interactions between the partner sites, create added value, and strengthen DKTK's overall research impact. The program supports innovative, collaborative activities in basic and preclinical research, infrastructure development, and multicenter investigator‐initiated trials (IITs), which are identified in a highly competitive evaluation and selection process involving the SAB. So far, a total of 20 projects and IITs have been supported within DKTK's Joint Funding Program, each involving at least three and often all eight partner sites. Ongoing studies are listed in Table 2 with two recently initiated DKTK‐wide strategic initiatives in the fields of imaging and surgery. DKTK's Joint Funding Program has been crucial for increasing DKTK's productivity and is expected to leverage further funding as well as further innovative and larger scale clinical trials.

**Table 2:** DKTK's ongoing joint funding projects and consortium‐wide strategic initiatives

Title of project or IIT	Number of participating partner sites
AMPLIFY‐NEOVAC Trial: AMPLIFYing NEOepitope‐specific VACcine Responses in progressive diffuse gliomas	7
DKTK MASTER Program: Cross‐Entity Molecular Stratification Program for Evaluation of Individualized, Biology‐Driven Therapy in Younger Adults with Advanced‐Stage Cancer and Patients with Rare Tumors (MASTER, Molecularly Aided Stratification for Tumor ERadication)	All
ImmuNeo MASTER: Impact of mutational pathways on immune environment, neoantigens and tumor rejection for the development of comprehensive biomarker panels and combinatorial approaches in cancer immunotherapy	All
iVacALL Trial: Prospective phase I/II study: Patient‐individualized peptide vaccination based on whole exome sequencing with adjuvant GM‐CSF and IFNα in children with relapsed acute lymphoblastic leukemia	5
NonCoMs in Cancer Genomes: Identifying and Understanding Non‐coding Mutations in Cancer Genomes	4
Overcoming Therapy Resistance in Pancreatic Cancer: Development of combinatorial therapies targeting primary and secondary drug resistance of pancreatic cancer subtypes	5
Ga‐68‐PSMA‐11 in High‐Risk Prostate Cancer Trial: An open‐label, single‐arm, rater‐blinded, multicenter phase 1/2 study to assess safety and diagnostic accuracy and radiotherapeutic implications of pre‐operative Ga‐68‐PSMA‐11 PET/CT imaging in comparison to histopathology, in newly‐diagnosed prostate cancer (PCA) patients at high risk for metastasis, scheduled for radical prostatectomy (RP) with extended pelvic lymph node dissection (EPLND).	7
Targeting Myc: Therapeutic Targeting of MYC	5
Translation of Molecular‐Based Treatment Approaches in ALL: Functional modeling of molecular‐based treatment approaches in relapsed acute lymphoblastic leukemia	3
UniCAR NK Cells: Chimeric antigen receptor‐engineered natural killer cells as a universal cellular therapeutic for adoptive cancer immunotherapy	4
DKTK Joint Imaging Platform: Strategic initiative ‘Distributed IT Infrastructure for Multilateral Imaging Cohort Analysis’	All
DKTK Surgery: Strategic initiative ‘Surgical oncology in the era of precision medicine: Creation of an ultra‐high quality bio‐ and databank for identification of predictive markers for individualized surgical treatment of gastrointestinal tumors’	All

### 2.3. Research platforms and infrastructures

The DKTK has made strong efforts to set up cutting‐edge joint infrastructures, providing access to highly sophisticated technologies and services.

The *Clinical Communication Platform (CCP)* has been established as the central hub of DKTK for collecting and exchanging clinical data and biomaterials and thereby provides a bridge across disciplines and institutions. Located at partner‐site Frankfurt, the CCP office organizes task forces for clinical data management, a task force for biobanking, and a team of IT specialists to develop and implement information technology solutions. The CCP has begun to establish long‐term, systematic, smart, and flexible solutions for documenting clinical data, storing and handling biomaterial, and exchanging information among DKTK partner sites. Most importantly, a virtual, joint clinical and biomaterial database has been developed as an advanced IT backbone and interface at all partner sites (‘bridgeheads’). Its major purpose is to provide consortium‐wide access to high‐quality patient data, clinical trial information, and information about stored biomaterials. Currently, to further pursue translational clinical cancer research on a national scale, the unique DKTK‐CCP bridgehead structure is rolled out to non‐DKTK CCCs sponsored by the Deutsche Krebshilfe (German Cancer Aid) within their Comprehensive Cancer Center of Excellence program (which also includes all DKTK partner sites).

Within DKFZ's *Cancer Genome Sequencing and Proteome Analysis Platform*, a DKTK sequencing facility has been built into a national cancer sequencing core center, which is available to all DKTK scientists and beyond. The platform has facilitated the exchange of expertise among the different sites and created harmonized procedures and common standards, and also offers advanced bioinformatics services by one of the largest and most experienced clusters of biological data scientists. The facility has been instrumental in the stimulation and successful completion of multiple high‐impact research projects. Complementary to the genome sequencing core, a decentralized, harmonized Proteome Platform is currently being established within DKTK to bundle a wide range of proteomics expertise in the network and to make the respective technologies, equipments, and specialized expertises available to researchers of the consortium.

Twice weekly, consortium‐wide molecular tumor boards were established within DKTK based on the CCP and Genome Sequencing Platform. While immunohistochemistry, FISH, or NGS panel‐based molecular diagnostics for discussions in multidisciplinary tumor boards has become clinical routine during the past decade at all DKTK partner sites, DKTK paved the way in Germany for site‐overarching utilization of advanced molecular diagnostics including whole‐genome sequencing for cutting‐edge multicenter trials.

Further unique platforms have strengthened interinstitutional translational and clinical research in the field of radiation oncology and imaging, including all current German particle therapy units and a wide range of preclinical and clinical high‐field magnetic resonance imaging (MRI) und positron emission tomography (PET) units. The powerful RadPlanBio platform, hosted in Dresden and Heidelberg, has been specifically developed by DKTK data scientists for dose‐space‐time resolved data storage, analysis, and exchange over all partner sites. It has been instrumental in various clinical trials and reverse translation projects of DKTK. It is currently being integrated as a module into the CCP and has also been expanded to a number of non‐DKTK sites to allow for further enlarging the patient base for translational studies in radiation oncology and imaging. Additional core services include a GMP facility located in Tübingen for central academic production of clinical‐grade antibodies and (personalized) peptide vaccines which are already being used in several clinical trials across the consortium. A genetic screening and gene editing technology platform helps identify novel targets and develop therapeutic interventions.

### 2.4. Cooperation management of research programs and platforms

Altogether, more than 1000 researchers are contributing to DKTK's translational research. Effective tools facilitating cooperation between the scientists at the different centers have been developed, for example, scientific workshops, seminars, and an annual retreat of the entire DKTK where achievements and further goals of the translation center are presented and discussed. All programs and platforms within DKTK are represented by coordinators who in addition to the Steering Committee facilitate development and implementation of the overarching DKTK research strategy, both DKTK‐wide and at the individual partner sites.

### 2.5. Examples of major scientific highlights

A fundamental task of future cancer research will be to address the great complexity that molecular profiling will add to the taxonomy of cancer. DKTK aims to make major contributions in this research area, as can be illustrated best by two highly successful programs: INFORM and MASTER.

The INFORM registry study (INdividualized Therapy FOr Relapsed Malignancies in Childhood) offers comprehensive genomic analyses nationwide to all children with relapsing cancers and provides information about biomarker‐stratified treatment options (Worst *et al*., 2016). Patients are discussed in INFORM interdisciplinary tumor boards. This program is now interlinked with other European programs on personalized oncology in pediatric cancer and initiating a series of exploratory biomarker‐driven early clinical trials (INFORM‐2).

The aim of the DKTK MASTER program (Molecularly Aided Stratification for Tumor ERadication Research) is multifold (Horak *et al*., 2017): (a) offering optimal molecular diagnostics by performing exome and transcriptome sequencing in all DKTK adult patients under 50 years of age who have advanced‐stage cancers or in patients with rare cancers; (b) providing all relevant information on the molecular status of every tumor as a stratification tool for treatment selection in individual patients; (c) developing a bioinformatics and systems medicine extension that incorporates bioinformatics predictions and experimental validation in addition to supporting clinical decision making through a physician interface for the interpretation of molecular data; and (d) implementing a program for anticancer targets to fund, support, and coordinate access to novel anticancer agents in IITs. MASTER patients from all partner sites are discussed in a consortium‐wide molecular tumor board, also providing excellent training for young clinician scientists. Furthermore, DKTK MASTER has triggered several, mostly multi‐institutional translational research activities and IITs in the consortium.

A similar approach was successfully applied in several Joint Funding projects in which novel personalized treatment strategies for patients with high‐risk glioma, acute leukemia, and lung cancer have been defined. DKTK is currently extending this paradigmatic approach to more advanced formats of clinical studies. One example is a study of relapsing EGFR‐mutated lung cancer, which involves DKTK centers, centers of the German Center for Lung Research (DZL), the national AIO study group, and four centers from France. This also demonstrates the successful interaction with another German Center of Health Research. Another example is the molecularly stratified umbrella trial NCT Neuro Master Match (N^2^M^2^), which has started recruiting adult patients with glioma at all DKTK sites (Pfaff *et al*., 2018; Wick *et al*., 2019). The clinical impact of this research approach is demonstrated by major contributions, which DKTK research could make to novel tumor classification schemes, as for example, the WHO Classification of Brain Tumors (Louis *et al*., 2016) or EANO guidelines for the diagnosis and treatment of adult astrocytic and oligodendroglial gliomas (Weller *et al*., 2017) and ependymal tumors (Ruda *et al*., 2018).

The DKTK Radiation Oncology Group, with participants from all partner sites, has identified clinically relevant biomarkers for individualizing radiotherapy of HNSCC in various multicenter studies. Biomarkers are being validated in prospective clinical studies, and prospective intervention studies are in preparation. These activities have been facilitated by DKTK's unique RadPlanBio platform, study center, and medicolegal unit.

Further innovative early clinical trials (see https://dktk.dkfz.de/en/research/clinical-trials for an overview), performed within the Joint Funding Program and beyond, include peptide vaccination in patients with leukemia, breast cancer, and brain cancer (including academic GMP production of personalized peptide vaccines), the use and validation of demethylating substances as therapeutic agents across cancer entities, and diagnostic PSMA (prostate‐specific membrane antigen) radiopharmaceuticals for prostate carcinoma imaging.

Research progress within DKTK has been reported in more than 2800 ISI‐cited articles, of which more than 540 were published in journals with impact factors above 10 (as of October 1st, 2018). Thus, DKTK has been highly successful in positioning itself as an important force for translational cancer research in Germany.

## 3. Novel career and training opportunities in translational oncology

As many other countries worldwide, Germany is facing the general challenge to attract young physicians and scientists into academic careers. Several scientific societies and committees, including the high‐level national Forum Health Research under the auspices of the Federal Ministry of Education and Research, have thoroughly analyzed the situation during the past years. Key recommendations include major investments into training and career perspectives of clinician and medical scientists. The German Centers for Health Research, including DKTK, are among the major addressees for this call for action.

### 3.1. DKTK professorships and young investigator groups

The strong network research environment as well as the increasing national and international visibility of DKTK attracts an increasing number of high potential trainees and leading advanced clinician and medical scientists to apply for open positions at DKTK partner sites. As a unique asset, DKTK can offer attractive long‐term career perspectives in the field of translational oncology. So far, 11 new joint DKTK full professors have been appointed who are working with a DKTK budget at the joint DKTK translation centers at all partner sites. These top‐class researchers are significantly shaping DKTK's progress in translational oncology research. DKTK has further invested great effort in attracting young talents to establish Young Investigator Groups. The DKTK's funding structure makes it possible to offer a tenure option, which is highly attractive for young scientists interested in translational research, and provide long‐term career perspectives for them. So far, eight new research units for young scientists have been established in the consortium.

### 3.2. Training and education of clinician and medical scientists

DKTK is making major efforts in developing structures to support translational clinical research for physicians and scientists at all career levels. To promote training and education, the *DKTK School of Oncology* has been established, offering training programs for clinician and medical scientists. The school significantly promotes interactions between laboratory‐based researchers, medical scientists, and clinician scientists, thereby creating a new translational culture among the next generation of cancer researchers in Germany. The curriculum covers key aspects of translational and interdisciplinary clinical research, including programs on unmet medical need assessment, trial design, medicolegal, and patient‐centered approaches. Clinician scientists and medical scientists undergoing clinical training in multidisciplinary cancer diagnosis and treatment are granted the time to establish a research project and to apply for further funding for their research. Overall, the DKTK School of Oncology (SoO) brings together more than 150 junior researchers at all partner sites, providing them with access to local programs and the DKTK SoO education events. New formats for interaction based on e‐learning and e‐doing tools bring further benefit to the existing network. The International Summer School in Translational Cancer Research, jointly performed with Cancer Core Europe (previously with the Eurocan Platform), attracts up to 60 international clinician scientists and medical scientists annually by offering a 1‐week teaching course on cutting‐edge topics such as innovative aspects of cancer biology, translational research, preventive and clinical research, and the bridging of these research fields.

## 4. Future directions and european perspective

DKTK has emerged, within only few years, as the leading network for translational cancer research in Germany. Researchers within but also beyond DKTK consider this consortium as a role model for research networking on a national level. Synergies and added value have been achieved in several ways, for example, by offering access to unique technologies, establishing clinical databases, and developing standardized procedures. In this way, quality‐controlled workflows for multicenter preclinical and clinical trials and large cancer registries are available at all partner sites. This is particularly relevant in light of the increased cancer stratification into molecularly distinct subentities.

Of particular importance for the success of DKTK, however, has been the generation of strong team spirit and a stimulating site‐overarching translational ecosystem through effective and inclusive governance, combined with the unique setting offered by the long‐term institutional funding needed for strategic and sustainable translational programs and structures. DKTK will further build on these foundations primarily in three ways:

First, and most straightforward, it will use its research network to contribute to tackling the major challenges in translational cancer research (i.e., personalized molecular diagnosis and therapy, tumor heterogeneity and resistance, immunotherapy, early detection of cancer and minimal residual disease, and turning big data into smart data) by conducting new and innovative research projects.

Second, together with the other DZGs, overarching structures and horizontal research programs will be generated. Thus, strong and very innovative cross‐linking activities can be implemented, for example, related to aging, comorbidity, artificial intelligence, drug development, or prevention. Furthermore, the DZGs including DKTK can build effective interaction platforms with industry or with regulatory authorities to streamline and improve trial regulation.

Third, DKTK will cooperate intensely with other translational cancer research centers and networks on a national and international scale. Within Germany, a number of CCCs with a strong translational research focus have emerged over the past years, which are not member of DKTK. Most of them are funded by an excellence program of the German Cancer Aid. It will be important for DKTK as well as for these centers to interact closely for leveraging the national research potential in this field. One good example in this direction is the access to the CCP also for non‐DKTK centers, but further such open activities need to follow. Whether it will be possible to extent DKTK by new partner sites needs to be further explored with the federal and state governments.

With about 55 000 new cancer patients per year, a joint IT and biobank structure over the consortium, and an increasing number of biologically and clinically well‐characterized patient cohorts as well as a growing number of innovative IITs, DKTK offers important assets for translational cancer research in international partnerships, for example, on rare tumors or highly stratified trials requiring large patient cohorts which are beyond the capacity of national networks. Cancer Core Europe is a potential umbrella for bringing together such national networks (see Eggermont *et al*., 2019), an option that should be explored within the upcoming European research programs, particularly a potential mission in cancer (Celis and Pavalkis, 2017).

## Conflict of interest

In the past 5 years, Dr Baumann attended an advisory board meeting of MERCK KGaA (Darmstadt), for which the University of Dresden received a travel grant. He further received funding for his research projects and for educational grants to the University of Dresden by Teutopharma GmbH (2011‐2015), IBA (2016), Bayer AG (2016‐2018), Merck KGaA (2016‐2030), Medipan GmbH (2014‐2018). Dr Baumann, as former chair of OncoRay (Dresden) and present CEO and Scientific Chair of the German Cancer Research Center (DKFZ, Heidelberg), signed/s contracts for his institute(s) and for the staff for research funding and collaborations with a multitude of companies worldwide. For the German Cancer Research Center (DKFZ, Heidelberg) Dr Baumann is on the supervisory boards of HI‐STEM gGmbH (Heidelberg).

For the present study, Dr Baumann confirms that none of the above mentioned funding sources were involved in the study design or materials used, nor in the collection, analysis and interpretation of data nor in the writing of the paper.

## Author contributions

All authors contributed to the writing of this article.
